# Centiloid recommendations for clinical context‐of‐use from the AMYPAD consortium

**DOI:** 10.1002/alz.14336

**Published:** 2024-11-20

**Authors:** Lyduine E. Collij, Ariane Bollack, Renaud La Joie, Mahnaz Shekari, Santiago Bullich, Núria Roé‐Vellvé, Norman Koglin, Aleksandar Jovalekic, David Valléz Garciá, Alexander Drzezga, Valentina Garibotto, Andrew W. Stephens, Mark Battle, Christopher Buckley, Frederik Barkhof, Gill Farrar, Juan Domingo Gispert, AMYPAD consortium

**Affiliations:** ^1^ Department of Radiology & Nuclear Medicine, Amsterdam UMC Vrije Universiteit Amsterdam The Netherlands; ^2^ Brain Imaging Amsterdam Neuroscience Amsterdam The Netherlands; ^3^ Clinical Memory Research Unit, Department of Clinical Sciences Malmö, Faculty of Medicine Lund University Malmö Sweden; ^4^ GE Healthcare Chalfont St Giles Buckinghamshire UK; ^5^ Centre for Medical Image Computing University College London London UK; ^6^ Memory and Aging Center, Department of Neurology University of California San Francisco California USA; ^7^ Barcelonaβeta Brain Research Center Pasqual Maragall Foundation Wellington Barcelona Spain; ^8^ IMIM (Hospital del Mar Medical Research Institute) Barcelona Spain; ^9^ Universitat Pompeu Fabra Barcelona Spain; ^10^ Life Molecular Imaging GmbH Berlin Germany; ^11^ Department of Nuclear Medicine, Faculty of Medicine and University Hospital Cologne University of Cologne Cologne Germany; ^12^ German Center for Neurodegenerative Diseases (DZNE) Bonn Germany; ^13^ Institute of Neuroscience and Medicine (INM‐2), Molecular Organization of the Brain, Forschungszentrum Jülich Germany; ^14^ Division of Nuclear Medicine and Molecular Imaging University Hospitals of Geneva Geneva Switzerland; ^15^ Department of Radiology and Medical Informatics University of Geneva Geneva Switzerland; ^16^ CIBM Center for Biomedical Imaging Lausanne Zwitserland; ^17^ Queen Square Institute of Neurology University College London London UK; ^18^ CIBER Bioingeniería, Biomateriales y Nanomedicina (CIBER‐BBN) Madrid Spain

**Keywords:** Amyloid‐β, Centiloid quantification, clinical trials, positron emission tomography

## Abstract

Amyloid‐PET quantification through the tracer‐independent Centiloid (CL) scale has emerged as an essential tool for the accurate measurement of amyloid‐β (Aβ) pathology in Alzheimer's disease (AD) patients. The AMYPAD consortium set out to integrate existing literature and recent work from the consortium to provide clinical context‐of‐use recommendations for the CL scale. Compared to histopathology, visual reads, and cerebrospinal fluid, CL quantification accurately reflects the amount of AD pathology. With high certainty, a CL value below 10 excludes the presence of Aβ pathology, while a value above 30 corresponds well with pathological amounts. Values falling in between these two cutoffs (“intermediate range”) are related to an increased risk of disease progression. Together, CL quantification is a valuable adjunct to visual assessments of amyloid‐PET images. An abnormal amyloid biomarker assessment is a key criterion to determine eligibility for anti‐amyloid disease‐modifying therapies, and amyloid‐PET quantification can add further value by precisely monitoring amyloid clearance, and hence guiding patient management decisions.

**Highlights:**

- Centiloid (CL) quantification robustly reflects of the amount of Aβ pathology.
- CL < 10/CL > 30 reflects Aβ‐negativity/positivity thresholds with high certainty.
- CL quantification is a valuable adjunct to visual assessments of amyloid‐PET.
- CL quantification can support trial design and treatment management.
- CL quantification could support the identification of early or emerging Aβ pathology.

## 1 INTRODUCTION

Biomarker quantification has emerged as a pivotal tool in the management of various diseases, allowing for precise measurements of critical parameters for clinical decision‐making and the development of novel drugs. In Alzheimer's disease (AD), the ability to quantify its primary pathological hallmark amyloid‐β (Aβ) through amyloid positron emission tomography (PET) imaging is a central tool for improving diagnostic accuracy and patient management.^1^, ^2^, ^3^ The emerging era of anti‐amyloid therapies investigated monoclonal antibodies such as aducanumab,^4^ gantenerumab,^5^ lecanemab^6^, ^7^ and donanemab,^8^ and relied on amyloid‐PET for patient selection, evaluation of target‐engagement, and assessment of drug effectiveness. In the Phase‐III study of donanemab, amyloid‐PET quantification was additionally utilized for making end‐of‐treatment decisions. Therefore, this imaging technique serves as a key biomarker guiding therapeutic strategies and could become an integrated part of clinical routine.

Until recently, the most common amyloid‐PET metric was the standardized uptake value ratio (SUVr) with thresholds specific to each tracer and processing pipeline. The Centiloid (CL) approach provides a universal means of calibrating SUVr measures of Aβ deposits into a tracer and quantification method‐independent unbounded scale.^9^ This quantification in “absolute” units can be leveraged into the definition of thresholds differentiating stages of AD pathology.^10^, ^11^, ^12^, ^13^, ^14^, ^15^, ^16^ However, considering the abundance of literature regarding CL cutoffs, disease stages, and their utility, there is a need for a structured and detailed overview to further the interpretability of the CL metric along the AD continuum and its clinical use. This work aimed to analyze and integrate the available knowledge of CL quantification and provide context‐of‐use recommendations for the implementation of the CL scale in clinical practice. These efforts have been collected and submitted as an application for a Biomarker Qualification Opinion (BQO) to the European Medicines Agency (EMA) on behalf of the Amyloid Imaging to Prevent AD (AMYPAD) consortium (www.amypad.eu). A draft opinion was adopted by the Committee for Medicinal Products for Human Use (CHMP) and published (EMADOC‐1700519818‐1200791).

## 2 THE CENTILOID SCALE: DEFINITION AND PROCESSING

**FIGURE 1:**
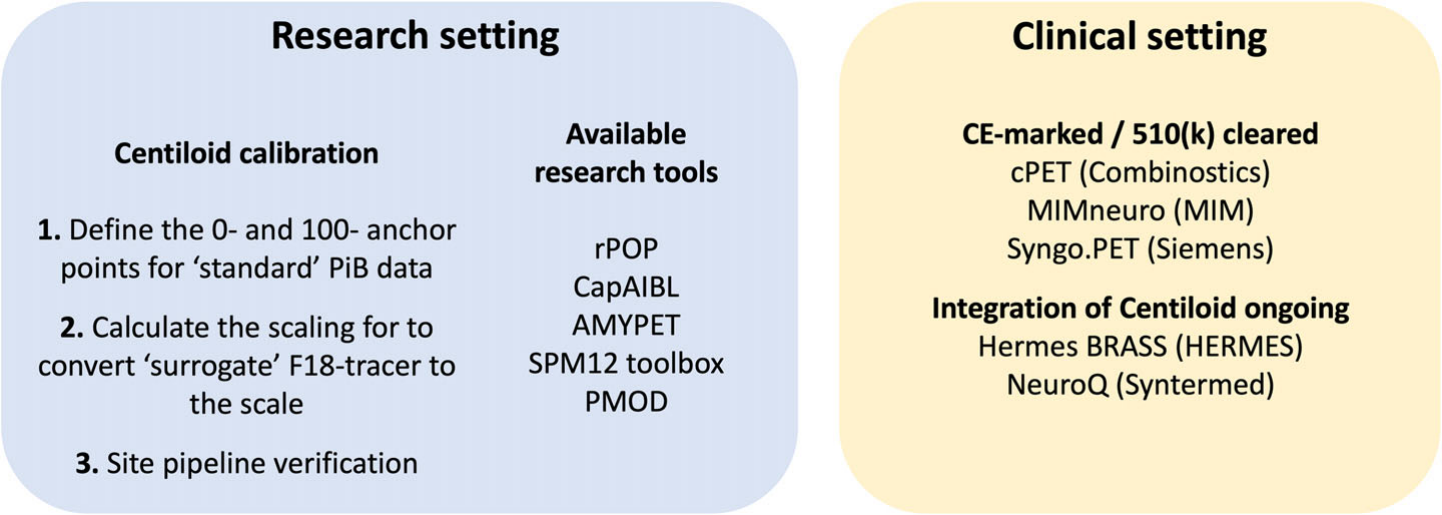
Overview of Centiloid implementation and available software in research and clinical settings. PiB, Pittsburgh compound B.

**FIGURE 2:**
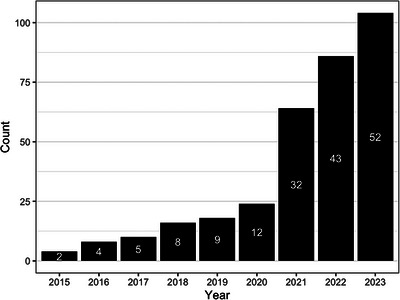
Centiloid publications in PubMed (search performed on the 01/01/24).

Amyloid‐PET quantification is commonly performed by normalizing the PET signal in a target region‐of‐interest (ROI) by the PET signal in a reference ROI supposedly free of specific tracer binding and summarized in the SUVr metric. Across the three regulatory approved radiotracers ([^18^F]florbetapir,^17^ [^18^F]florbetaben,^18^ [^18^F]flutemetamol^19^), differences in tracer binding and variability in image processing methodologies amplified in multi‐center studies led to the conceptualization of the tracer‐independent CL scale.^9^ More specifically, the CL method allows the transformation of amyloid PET data acquired at individual sites using the individual settings and processing into standard CL units using a two‐step scaling process. The key principle involves calibrating the SUVr of 18F‐labeled tracers to match those of [^11^C]Pittsburgh compound B (PiB), which are then transformed into the CL scale. The scale is anchored to the mean amyloid load of a reference young healthy control group (CL = 0) and to the mean amyloid load of a typical mild‐moderate AD population (CL = 100), available through the open‐source Global Alzheimer's Association Interactive Network (GAAIN) reference dataset (https://gaain.org/centiloid‐project), but values may be below 0 and above 100. The local processing pipeline is validated using specific quantitative criteria against the open‐source GAAIN dataset. Importantly, these calibration steps should be performed according to the guidelines mentioned in Klunk et al.^9^ for scientific use. For further clinical implementation, several EMA (CE‐marked) and/or Food and Drug Administration (FDA; 510[k])‐cleared softwares including the CL metric are (becoming) available (Figure 1, see also Table S1) and the package inserts of the amyloid PET tracers were amended in Europe to include quantification as an adjunct to the visual read (VR). Given its possible wide applicability and inherent generalizability, 159 papers have been published mentioning the CL scale since its first introduction in 2015 (Figure 2).

## 3 CENTILOID REFLECTS THE DEGREE OF AMYLOID PATHOLOGY

In the clinical routine, information regarding the degree of amyloid pathology is often omitted and reduced to a binary measure based on PET visual assessment, in line with current FDA and the initial EMA labels of the amyloid PET tracers. A major advantage of quantification is its ability to provide information on the amount of pathology, which could be reflective of different disease stages. In this section, existing CL thresholds established from gold (post mortem) and clinical standards (cerebrospinal fluid [CSF] and VR) are discussed.

### 3.1 Centiloid compared to the gold standard (post mortem)

Histopathological studies are the gold standard to establish the presence and amount of amyloid pathology. In most cases, these studies rely on the maximum neuritic plaque density found in specific neocortical areas defined using the Consortium to Establish a Registry for Alzheimer's Disease (CERAD).^20^ CERAD classifies neuritic plaque density into four categories: none, sparse, moderate, or frequent. The distinction between none‐to‐sparse and moderate‐to‐frequent as being Aβ‐negative or Aβ‐positive by pathology was used in the Phase‐III validation studies of the F‐18 amyloid tracers.^17^, ^18^, ^19^ Optimal thresholds to differentiate between none and sparse‐to‐frequent plaques have been suggested as 9.6 CL by Amadoru et al.^10^ and 12.2 CL by La Joie et al.^11^ Differentiating none‐to‐sparse versus moderate‐to‐frequent plaque density resulted in a wider range of optimal CL thresholds, from 12 to 35 CL.^10^, ^11^, ^21^, ^22^, ^23^, ^24^ A stricter approach focusing on none‐to‐moderate versus frequent amyloid plaques resulted in a threshold of 32.4 CL.^11^ In the same study, La Joie et al.^11^ also used Thal phases representing the spatial extent of Aβ pathology^25^ to propose thresholds of 12.0 and 23.5 CL to discriminate phases 0 to 1 versus 2 to 5 and 0 to 2 versus 3 to 5, respectively. Finally, using AD neuropathologic change (ADNC)—a combination of CERAD, Thal phases, and Braak tau stages^26^—La Joie et al.^11^ and Amadoru et al.^10^ found distinct thresholds (24.4 vs 49.4 CL, respectively) to detect intermediate‐to‐high versus none‐to‐low ADNC levels. The relatively high 49.4 CL threshold could be explained by the limited number of subjects with a baseline load between 25 and 50 CL and the fact that “likely AD” required a tau Braak III–IV positivity. Thus, the defined 50 CL cutoff might be more reflective of a level of Aβ pathology associated with established isocortical tau burden.

Despite variability in thresholds, these results suggest that amyloid pathology can be reliably excluded under 10 CL. In contrast, the cutoff for inclusion of amyloid pathology remains somewhat unclear, as postmortem data‐derived cutoffs fall between 12 and 50 CL, depending on included populations, neuropathological scores investigated, and variable intervals between PET acquisition and date of death. Studies comparing the CL scale to clinical measures provide further insights for ruling in Aβ pathology.[Fig alz14336-fig-0003]

### 3.2 Centiloid compared to clinically used reference standards (CSF and visual reads)

In clinical practice, AD pathology is commonly evaluated through VR of amyloid‐PET scans or analysis of CSF biomarkers, such as Aβ_42_, phosphorylated tau (pTau), and total tau (tTau).

The binary VR methods of the approved amyloid‐PET tracers were validated as part of their pivotal histopathology Phase‐III studies in end‐of‐life subjects. Studies using VR as the reference standard have reported a wide range of cutoffs reflecting Aβ‐positivity (17 to 42 CL).^2^, ^13^, ^27^, ^28^, ^29^, ^30^ This is most likely due to differences in reader experience, particularly regarding the assessment of more challenging scans displaying early amyloid‐PET uptake, substantial differences in the populations studied, with the number of preclinical individuals being limited or even absent in most VR studies, and differences in radiotracer kinetics. Nonetheless, most VR‐based thresholds were estimated to range between 21 and 26 CL.^10^, ^13^, ^31^ Expert readers could assess scans as visually positive down to a level of 17 CL and consistently at 25 CL.^13^, ^15^

Compared to CSF Aβ_42_, which is expected to change earlier in the AD continuum, Salvadó et al.^15^ proposed a cutoff of 12.1 CL to detect early amyloid abnormalities, which is in high agreement with the previously mentioned neuropathological studies.^10^, ^11^ In addition, a second threshold of approximately 30 CL was identified to indicate the presence of established pathology based on pTau, tTau, and their ratio with Aβ_42_. Importantly, this cutoff was highly consistent (28.6 to 29.7) across these CSF biomarkers reflective of established AD pathology Aβ_42_.

Collectively, these findings indicate that at an individual level, 30 CL is a conservative threshold above which there is a high certainty that a relevant amount of amyloid pathology is established.

### 3.3 CL in the “intermediate range”

**FIGURE 3 alz14336-fig-0003:**
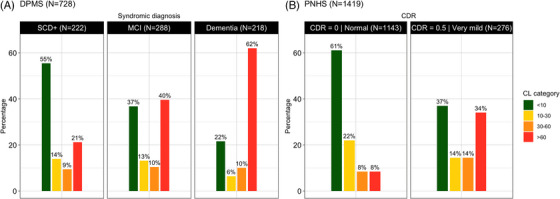
AMYPAD participants categorized by cognitive stage and baseline CL. Bar plots illustrate the distribution of CL groups across two studies, (A) DPMS and (B) PNHS, from the AMYPAD consortium. Subjects were categorized into four groups based on their baseline amyloid burden: <10 CL (no amyloid pathology), 10 to 30 CL (“intermediate range,” evolving amyloid pathology), 30 to 60 CL (established amyloid pathology with increased prevalence of tau positivity), and >60 CL (established amyloid pathology with high certainty of tau‐PET positivity). AMYPAD DPMS and PNHS datasets are publicly available (https://amypad.eu/data/). AMYPAD, Amyloid Imaging to Prevent Alzheimer's Disease; CDR, Clinical Dementia Rating; CL, Centiloid; DPMS, Diagnostic and Patient Management Study; MCI, mild cognitive impairment; PNHS, Prognostic and Natural History Study; SCD+, subjective cognitive decline plus.

The window between 10 and 30 CL units can be regarded as an “intermediate range,” indicative of an evolving pathology trending towards positivity. In the AMYPAD Diagnostic and Patient Management Study (DPMS) dataset, with participants whose features are consistent with those expected in a memory clinic population,^3^ around 10% of participants were in that CL band (Figure 3A). Nonetheless, the percentage of intermediate range participants is substantially higher in preclinical AD populations. For example, the AMYPAD Prognostic and Natural History Study (PNHS) reported 22% of individuals with a CL between 10 and 30 units in their primarily cognitively unimpaired cohort above 50 years of age, which is the current target population of early secondary prevention studies like the AHEAD study (Figure 3B).^32^ As such, characterizing the CL intermediate range and its associated risk for future disease progression is key.

#### 3.3.1 Centiloid thresholds and disease progression

A growing number of studies have investigated potential cutoffs in the 10 to 30 CL intermediate range to enable the identification of individuals with an increased risk of disease progression. In the earliest stages of AD, Farrell et al. observed an optimal threshold to predict future significant Aβ accumulation ranged from 15 to 17.5 CL in cognitively unimpaired participants across the Australian Imaging, Biomarker and Lifestyle (AIBL), Harvard Aging Brain Study (HABS), and AD Neuroimaging Initiative (ADNI) cohorts.^33^ This finding is consistent with the commonly described peak in rate of change before reaching amyloid positivity,^33^, ^34^ and with the reliable worsening estimate of 19 CL determined by Jack and colleagues, which reflects the cut point beyond which the amyloid rate of change increases reliably beyond baseline variation in the measure.^35^ At the higher end of the intermediate range, a CL of 26 was found to best predict progression to dementia.^27^

Beyond the intermediate range, two studies highlighted a significant rise in the prevalence of tau‐PET positivity within the 40 to 70 CL range across ROIs reflecting early to established tau burden.^16^, ^36^ Notably, in cognitively unimpaired individuals with CL levels below 50, no tau‐PET positivity was observed in the neocortex.^16^ Tau‐related amyloid levels could be important for patient characterization and selection in future clinical trials. For example, the success of the donanemab TRAILBLAZER‐ALZ2 study has been accredited to their participant stratification‐based tau burden, selecting specifically those early AD patients that were Aβ positive (CL > 37) and had intermediate tau PET burden.^8^ Recently at the Clinical Trials on AD (CTAD) conference, it was demonstrated that this potential high‐benefit group could be accurately selected based on CL quantification, with a reported cut point of 60 CL.^37^ Taken together, CL provides a tracer‐independent measurement of the amount of Aβ pathology. This can be used to stage the disease, to identify those in the early stages who are at risk for progression, and to identify individuals who can benefit the most from disease‐modifying treatments. These risk‐stratification approaches in combination with other commonly available clinical biomarkers could also support longitudinal monitoring efforts at the individual level, but more studies in such populations and with other outcome measures are needed to further elucidate the CL intermediate range.

## 4 ROBUSTNESS OF THE CENTILOID SCALE

The AMYPAD consortium was uniquely positioned to assess the robustness of the CL metric due to its multi‐site nature and collaborations with the EARL (https://earl.eanm.org/), the initiative of the European Association of Nuclear Medicine (EANM), to harmonize quantification in nuclear medicine imaging. To optimally interpret CL values and their associated thresholds, it is critical to consider methodological steps that could impact the precision of the metric. The importance of quantifying PET measurements and their uncertainty have been highlighted in the latest Radiological Society of North America (RSNA) Quantitative Imaging Biomarkers Alliance (QIBA) profile.^38^ The principles in this paper are discussed with respect to the SUVr measure but are equally applicable to the CL measure.

### 4.1 Sensitivity to pipeline design

While the standard CL pipeline as described by Klunk and colleagues is based on the Statistical Parametric Mapping (SPM), an abundance of alternative pipelines are available.^9^ Within the multi‐site AMYPAD consortium, the sensitivity of CL quantification to pipeline design, age, atrophy, and image resolution was evaluated by a head‐to‐head comparison of 32 calibrated CL pipelines covering the typical pipeline design options. In this experiment, four factors were tested: the reference region (RR; whole cerebellum [the primary one], cerebellum grey matter, pons, and cerebellum + brainstem), the analysis space (standard Montreal Neurological Institute [MNI] space vs native space), and definition of target and reference volumes of interest (VOIs; GAAIN VOIs or subject‐based segmentations of gray and white matter). Using 533 participants from the AMYPAD‐DPMS dataset (*N*_[18F]flutemetamol _= 207, *N*_[18F]florbetaben _= 123) and matched individuals from the ADNI database (*N*_[18F]florbetapir _= 203), generalized estimating equations (GEEs) were used to assess the impact on CL values of the various pipeline design factors.^39^ Overall, CL quantification was robust against tracer, and differences in image resolution while using the standard CL pipeline recommended by Klunk et al.^9^ Moreover, CL quantification was minimally affected by atrophy in the participants with an average CL of 90, and therefore these differences have no impact on the clinical diagnosis. Importantly, the pons reference region yielded significantly lower CL values as a consequence of age‐related white matter uptake, particularly in participants with lower amyloid burden.^40^ Within‐pipeline 95% confidence intervals ranged from ± 3.3 to ± 4.0 CL between 12 and 24 CL, respectively.

### 4.2 PET‐only versus MRI‐based pipeline comparison

The need for a magnetic resonance imaging (MRI) scan for CL quantification, as proposed in the original pipeline by Klunk and colleagues, could limit its clinical implementation. Consequently, several PET‐only pipelines have been developed, which do not rely on MR for accurate registration.^41^, ^42^ For example, the Imaging Dementia—Evidence for Amyloid Scanning (IDEAS) study developed a robust PET‐Only Processing (rPOP) pipeline. Thousands of amyloid‐PET scans from this study were quantified and similar agreements against VR and the standard CL pipeline were obtained.^41^ The AMYPAD consortium performed a head‐to‐head study, comparing the standard PET‐MR pipeline to the AMYPYPE PET‐only pipeline^43^, ^44^ to ensure consistent results regardless of the design. An analysis using 283 [^18^F]flutemetamol scans showed a strong correlation (*R* ^2 ^= 0.97) between the two pipelines and no amyloid burden‐dependent systematic bias. There was a low mean absolute deviation (5.6 ± 4.7 CL) that fell within the noise levels computed in those datasets. Other PET‐only CL pipelines have been implemented with excellent performance against MR‐based methods, such as the CapAIBL^45^ or the Non‐negative Matrix Factorization method.^14^

### 4.3 Impact of error propagation on Centiloid conversion equations

We also investigated the impact of measurement uncertainty or error propagation in the initial development of the CL conversion equations using a simulation‐based design.^46^ This was done by adding heteroscedastic Gaussian noise with a larger standard deviation (SD) for higher CL values to 10,000 bootstrap simulations and generating the corresponding CL calibration equation for each simulation. Simulated noise was obtained for each tracer separately and defined as the SD of the error measured in healthy volunteers (0 CL) or AD subjects (100 CL). Simulated noise at intermediate values between 0 and 100 CL was obtained by linear interpolation. A jackknife approach was used to confirm the results obtained from the bootstrap simulations. Overall, in the lower end of the scale, the maximum bias due to error propagation had a small impact on CL measurements (<3.5 CL), while it increased in average up to 8 CL for higher CL values (between 75 and 100 CL). Importantly, the increase in maximum error in the higher range of the scale (≈80 CL) would not have affected classification at the individual level for most of the cutoffs described so far.

### 4.4 Reliable Aβ accumulation

Assessment of test–retest variability and error propagation of the CL metric is needed to determine what change in CL constitutes a reliable accumulation. The CL metric has been proposed as a useful measure to track amyloid pathology over time and help determine if a subject is accumulating amyloid at a higher pace than healthy elderly individuals. Using data from 1032 participants from the AMYPAD PNHS and Insight46 who underwent [^18^F]flutemetamol, [^18^F]florbetaben, or [^18^F]florbetapir amyloid‐PET imaging, a normative strategy was used to define reliable accumulation by estimating the 95th percentile of longitudinal measurements in subpopulations (*N*_PNHS _= 101/750, *N*_Insight46 _= 35/382) expected to remain stable over time. Reliable accumulation was estimated to occur at > 3.0 CL/year.^47^ Additional evidence is provided through the placebo groups in recent anti‐amyloid trials, which reported rates of amyloid accumulation at a similar magnitude (with baseline amyloid loads between 75 and 101 CL, Figure S1). For example, in the GRADUATE 1 and 2 trials of gantenerumab, the accumulation was approximately 4 CL/year.^5^ In the case of aducanumab (high dose), the rates were −0.6 and 2.2 CL/year in the ENGAGE and EMERGE trials, respectively.^4^ For lecanemab's Clarity‐AD trial, the rate was 2.4 CL/year,^37^ while in the donanemab TRAILBLAZER ALZ2 trial (low/medium tau), it was 0.1 CL/year.^8^ Following amyloid clearance in the donanemab trial, a re‐accumulation rate of 2.8 CL/year was observed.^48^

Of note, in the PNHS, rates of amyloid accumulation from linear mixed‐effect models were tracer independent and lower for apolipoprotein E (*APOE*) *ε*4 noncarriers and for subjects with higher levels of education. Importantly, these results were obtained in a harmonized and curated research cohort. Reliable accumulation at the individual level in real‐life clinical settings could be greater.

## 5 PROPOSED CONTEXT‐OF‐USE

**FIGURE 4:**
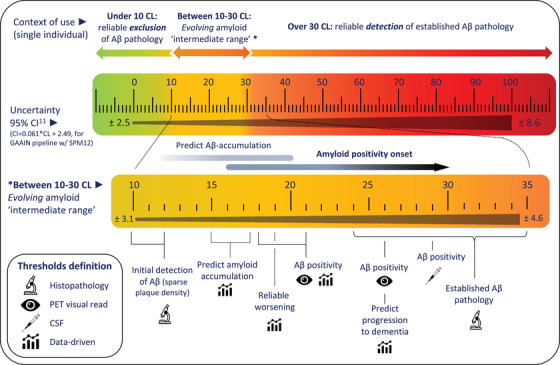
Overview of Centiloid scale interpretation. An illustrative figure summarizing the main studies regarding CL‐based cut points to reliability exclude (<10 CL) and include (>30 CL) Aβ‐pathology at the individual level. In addition, the 95% confidence interval across the scale is provided and detailed cutoffs in the “intermediate range” are highlighted. Aβ, amyloid‐beta; CL, Centiloid; CI, confidence interval; CSF, cerebrospinal fluid; GAAIN, Global Alzheimer's Association Interactive Network; PET, positron emission tomography; SPM, Statistical Parametric Mapping.

Taken together, the CL scale provides a valuable approach to attain a standardized and generalizable measure of amyloid burden across tracers and centers. Based on the above, we conclude that CL quantification accurately reflects the amount of AD pathology with a CL value of <10 excluding the presence of any Aβ pathology, while a value of >30 is a conservative estimate of amyloid positivity. Importantly, these proposed cut points reflect the optimal sensitivity and specificity of the metric, respectively, providing high certainty at the individual level (Figure 4). The full distribution of amyloid‐positive cut points mentioned can be found in Figure S2. Below we provide specific user guidelines for different scenarios:

1. **Centiloid quantification is a valuable adjunct to visual assessments of amyloid‐PET images to achieve high certainty** **regarding the presence or absence of Aβ pathology**.
  This is particularly the case for equivocal scans or for readers with less experience, as recently demonstrated in a prospective study of challenging clinical cases, showing a significant increase in reader agreement and confidence after disclosure of quantitative results.^49^ If CL burden is discordant with the visual assessment or falls in between the reliable inclusion and exclusion criteria previously defined (“intermediate range”; in between 10 and 30 CL), readers are encouraged to review the scan for the following signs: focal uptake, brain atrophy, or poor scan quality. Of note, in a clinical setting, individuals with a CL in the intermediate range are a minority (Figure 3A). It is also important to note that both assessment approaches have inherent limitations, as visual reads can be dependent on reader experience, and (CL) quantification is sensitive to deviations in acquisition protocols and processing errors. As such, we recommend the joint use of both assessments to minimize the number of Aβ status misclassifications.
2. **Anti‐amyloid disease modifying therapies: patient inclusion**
  Drug initiation of anti‐amyloid monoclonal antibodies such as lecanemab and donanemab requires high specificity of Aβ status, as serious side effects should only be risked in case of potential treatment benefit. In this context, patients with objective cognitive impairment as determined by a standard neuropsychological evaluation and an abnormal Aβ biomarker could be eligible for these anti‐amyloid disease‐modifying therapies. An amyloid burden of 30 CL or above could be considered the Aβ‐positivity threshold with high certainty (ie, a more conservative threshold) at the individual level. This 30 CL cut point takes into the account the 95% confidence intervals reflecting the uncertainty in CL quantification.
  On the other hand, trials focused on early, primary, or alternative interventions such as changes in lifestyle potentially require optimal sensitivity. As such, the 10 CL threshold (ie, a more liberal threshold) could be implemented to screen out subjects with higher certainty of being Aβ‐negative.
3. **The degree of Aβ clearance after treatment with anti‐amyloid disease‐modifying therapies can be reliably measured with the Centiloid method**.
  CL quantification could support significant management changes such as cessation of anti‐amyloid therapy once full clearance (ie, amyloid negativity) has been observed.^8^, ^50^ Similar criteria could be applied to identify nonresponders after a given treatment period. Relevant to this, anti‐amyloid drugs have reported reductions in CL units in the range of 60 to 85 CL, much higher than the uncertainty in CL quantification although treatment cessation decisions might be between 11 and 25 CL^8^ and hence the value of knowing the reliability of the CL pipelines at these measures is valuable.
4. **Centiloid quantification could support the identification of early or emerging Aβ pathology, as values that fall within the intermediate range are associated with an increased risk of disease progression**.
  Particularly, values above 15 CL have been associated with significant Aβ accumulation towards established Aβ pathology. In addition, reliable accumulation is between 3 and 5 CL per year, depending on the heterogeneity of the cohort, while test–retest and measurement error at the lower end of the scale is between 2.5 and 3.5 CL. Together, CL can be used to include patients in early secondary prevention studies.

## 6 DEVELOPMENTS AND IMPLEMENTATION

Other clinical applications of the CL scale are currently being investigated. In addition to supporting the detection of amyloid burden and achieving high confidence of Aβ status based on visual assessment, CL quantification could also provide relevant information to support differential diagnosis. A recent study in a mixed memory clinic population illustrated that visual amyloid‐positivity was associated with a wide range of CL units across clinical stages. Importantly, CL quantification was associated with the primary etiological diagnosis after correction for the clinical stage, with AD patients displaying the highest amyloid burden, followed by dementia with Lewy bodies and cerebrovascular disease.^2^ These results also support the notion that CL levels lower than expected, based on clinical stage, might be indicative of co‐pathology rather than the primary etiology underlying cognitive decline.

**FIGURE 5:**
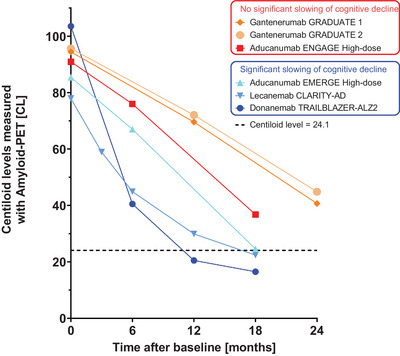
Amyloid‐β removal profiles for Phase‐III trials of aducanumab, donanemab, gantenerumab, and lecanemab as measured by Centiloid (CL). Sample sizes in the treatment arms varied for the individual trials and were at the last visit: *N *= 614 for donanemab, *N *= 210 for lecanemab, *N *= 50 (Graduate 1) and *N *= 41 (Graduate 2) for gantenerumab, and *N *= 109 (Emerge high‐dose) and *N *= 112 (Engage high‐dose) for aducanumab. Data points as reported in Sims et al. (2023), van Dyck et al. (2022), Bateman et al. (2023), and Budd Haeberlein et al. (2022). Placebo groups are not plotted and showed constant or slightly increasing amyloid levels over time, as expected. The dotted line represents 24.1 CL, the cutoff for amyloid‐negativity as defined within the GRADUATE 1 and 2 trials and implemented in the head‐to‐head studies of donanemab versus Aduhelm.

In the context of clinical trials and future routine implementation, further work is needed to assess how CL values could be used to further improve patient benefit–risk stratification and for monitoring an individual's response to therapy. Recently completed anti‐amyloid trials revealed a slowing of cognitive decline if full clearance of amyloid was obtained within 18 months (Figure 5). Determining successful amyloid clearance would allow discontinuation of treatment, reducing patient and facility burden, costs, and potential harmful side effects. The donanemab TRAILBLAZER‐ALZ2 Phase‐III trial already implemented this approach, switching patients to placebo if one PET scan revealed CL < 11, or if two consecutive PET scans had amyloid levels in between 11 and 25 CL (amyloid clearance defined at 24.1 CL).^8^ After anti‐Aβ therapy, CL estimates could also be used to assess potential re‐accumulation.^51^ In terms of risk stratification, future work is needed to elucidate to what extent continuous CL burden could be implemented to further inform on individual risk–benefit ratios. For example, results from the donanemab Phase‐III trial demonstrate that clinical benefit is reduced in patients with high tau burden (possibly associated with higher CL values; see Figure S3). Additional CL cutoffs could be implemented to estimate an individual's likelihood of tau‐PET burden. In addition, results presented at the CTAD 2023 conference suggest that high baseline CL in combination with other risk factors such as homozygous *APOE ε*4 carriership, cardiovascular risk factors, and the presence of microbleeds and/or superficial siderosis based on MRI brain imaging, is associated with risk of developing amyloid‐related imaging abnormalities (ARIA), the most common side effect of anti‐amyloid therapies.^52^, ^53^, ^54^

Finally, the rapid developments in plasma biomarker assays are revolutionizing the field of patient screening/selection for trials and disease‐modifying treatments.^55^ Blood‐based biomarkers are convenient for predicting amyloid positivity and to a certain extent tau burden.^56^ Considering the disease‐staging capacities of CL cut points, it is of great interest to determine whether CL could also be used in conjunction with plasma biomarkers to further optimize stratification and support risk–benefit considerations at the individual level.^57^

**Box 1: Centiloid quantification: A powerful tool, not a magic bullet**

**Strengths**:

The Centiloid (CL) method:

1. provides a global, semi‐quantitative measure of amyloid‐β burden as measured by positron emission tomography (PET) imaging;
2. improves the interpretability of amyloid‐PET values with clinically relevant 0 and 100 anchor points;
3. facilitates dataset sharing and merging across groups using different tracers and/or pipelines;
4. allows comparison of amyloid‐PET values across publications and clinical trials.

**Notes of caution**:

1. Similar to standard uptake value ratio (SUVr) values, CLs
  - depend on the quality of image acquisition and processing
  - can be inaccurate in case of atypical amyloid distribution affecting the reference region (eg, the elevated signal in the cerebellum) or the cortex (eg, signal limited to cortical areas outside the mask)
2. Rigorous steps are needed to validate the equation to convert original PET metrics (eg, SUVr) to CLs; equations are tracer/pipeline/acquisition time‐window specific.
3. CL transformation harmonizes the dynamic range across tracers, but not measurement error/noise

## 7 METHODOLOGICAL CONSIDERATIONS AND LIMITATIONS

The application of the CL scale, although a significant advancement in the standardization of amyloid PET quantification, still presents some outstanding challenges. One critical issue is to verify the thresholds across different populations, particularly those that are underrepresented in existing research, such as Black and Hispanic individuals.^58^ This is to ensure there are no recruitment biases in clinical and research settings. Thus, further studies in underrepresented populations are required to enhance the generalizability of CL‐based assessments.

Moreover, the precision of amyloid measurement is inherently linked to the dynamic range of the tracer used. Evidence suggests that the variance of CL values in young, cognitively normal individuals—expected to be amyloid‐negative—is somewhat higher for [^18^F]florbetapir compared to [^18^F]florbetaben and [^18^F]flutemetamol.^1^ The effect of tracer on cutoff determination is unknown, as proper head‐to‐head studies are lacking. Nonetheless, the studies presented in this review cover all three commonly used F18 radiotracers and [^11^C]PiB, and the consistent identification of cut points within the noise range across studies, tracers, and populations suggests that CL mitigates this effect properly. A more specific example comes from the Dominantly Inherited Alzheimer Network study, which showed that PiB and [^18^F]florbetapir differ in their ability to track treatment response using SUVr but not CL, consistent with the intended purpose of the CL method. Therefore, this study shows that the CL scale eliminates between‐tracer longitudinal differences even when changes are due to anti‐Aβ drug treatment, as opposed to disease progression alone.^59^ Future studies should determine the tracer‐associated bias of CL and determine whether cut point confidence intervals are tracer‐dependent.

In related fashion, CL quantification demonstrates similar results across different tracers at the group level, providing a reliable means of comparing amyloid burden in large‐scale studies. However, this apparent consistency could mask underlying discrepancies due to technical factors at the individual level. Further studies could investigate the CL variability across established research pipelines and regulatory‐approved software and the impact of clinical decision‐making at the single‐person level.

Finally, the CL approach provides a robust measure of the extent of amyloid burden due to its standardized target ROI.^9^ While this ROI encompasses the main cortical regions showing amyloid‐PET uptake, posterior/occipital regions are underrepresented or even excluded. As such, atypical patterns of amyloid‐PET signal could be missed using CL quantification,^60^, ^61^ further highlighting the importance to utilize quantification in conjunction with visual assessments.^44^

## 8 CONCLUSION

For the three approved FDA/EMA amyloid‐PET radiotracers, the CL scale is a robust, tracer‐independent, and validated metric reflecting the degree of amyloid pathology that is suitable to be used in clinical settings. At the individual level, a CL below 10 reliably excludes amyloid pathology, while a CL above 30 reliably detects abnormal accumulation. Values between these thresholds signify an intermediate range, indicating evolving levels of amyloid. The uncertainty of the CL metric (≈3 CL around 10 CL, up to 8 CL around 80 CL) should always be considered when interpreting the amyloid load against established thresholds and in longitudinal evaluations. In the future, CL quantification could be used for benefit–risk stratification, disease monitoring, and patient management. Notably, the use of amyloid‐PET quantification in the clinical routine as adjunct to the visual assessment is currently approved in Europe by the EMA and Medicines and Healthcare Products Regulatory Agency (MHRA). In the US, the FDA‐approved method for PET scan assessment is still based on VR only. This perspective review provides further evidence for its utility and global implementation.

## CONFLICT OF INTERESTS STATEMENT

Ariane Bollack is employed by GE HealthCare. Alexander Drzezga has received research support from Siemens Healthineers, Life Molecular Imaging, GE Healthcare, AVID Radiopharmaceuticals, Sofie, Eisai, Novartis/AAA, and Ariceum Therapeutics; has served as Speaker Honorary/Advisory Boards for Siemens Healthineers, Sanofi, GE Healthcare, Biogen, Novo Nordisk, Invicro, Novartis/AAA, and Bayer Vital; holds stock in Siemens Healthineers, Lantheus Holdings, Structured therapeutics, and ImmunoGen; and holds a patent for 18F‐JK‐PSMA‐7 (Patent No.: EP3765097A1; Date of patent: Jan 20, 2021). Aleksandar Jovalekic is employed by Life Molecular Imaging. Andrew W. Stephens is employed by Life Molecular Imaging. Christopher Buckley is employed by GE HealthCare. David Valléz Garciá has no relevant disclosures. Frederik Barkhof is supported by the NIHR biomedical research center at UCLH; serves as a steering committee or Data Safety Monitoring Board member for Biogen, Merck, Eisai, and Prothena; serves as an advisory board member for Combinostics and Scottish Brain Sciences; serves as a consultant for Roche, Celltrion, Rewind Therapeutics, Merck, and Bracco; has research agreements with ADDI, Merck, Biogen, GE Healthcare, and Roche; and is co‐founder and shareholder of Queen Square Analytics LTD. Gill Farrar is employed by GE HealthCare. Juan Domingo Gispert has received research support from GE HealthCare, Roche Diagnostics, and Hoffmann – La Roche; has received speaker/consulting fees from Roche Diagnostics, Philips Nederlands, Esteve, Biogen, and Life Molecular Imaging; and serves in the Molecular Neuroimaging Advisory Board of Prothena Biosciences. Lyduine E. Collij has received research support from GE Healthcare and Springer Healthcare (funded by Eli Lilly), both paid to the institution; Dr. Collij's salary is supported by an MSCA postdoctoral fellowship research grant (#101108819) and an Alzheimer Association Research Fellowship (AARF) grant (#23AARF‐1029663). Mark Battle is employed by GE HealthCare. Mahnaz Shekari has no relevant disclosures. Norman Koglin is employed by Life Molecular Imaging. Núria Roé‐Vellvé is employed by Life Molecular Imaging. Santiago Bullich is employed by Life Molecular Imaging. Renaud La Joie receives funding from NIH/NIA (P30‐AG062422, K99AG065501), the US Department of Defense, and the Alzheimer's Association (AARG‐22‐926899); and is an associate editor for *Alzheimer's Research & Therapy*. Valentina Garibotto is supported by the Swiss national science foundation (project n.320030_185028 and 320030_169876), the Aetas Foundation, the Schmidheiny Foundation, the Velux Foundation, the Fondation privée des HUG; and has received support for research and speakers’ fees from Siemens Healthineers, GE HealthCare, Janssen, and Novo Nordisk, all paid to the institution. Author disclosures are available in the Supporting information.

## Supporting information



Supporting information

Supporting information
