# Supplementary material for: Centiloid recommendations for clinical context‐of‐use from the AMYPAD consortium
Source: Alzheimers Dement. 2024 Nov 20;20(12):9037–48. doi: 10.1002/alz.14336 (PMC11667534; doi:10.1002/alz.14336)
Supplement: Supplementary file 1 — Supporting information [file ALZ-20-9037-s002.docx]

**Supplementary Tables and Figures**

**Supplementary Table-1. Overview of available software’s including Centiloid for clinical and research purposes**

| ***Software*** | ***Centiloid implementation*** | ***Open-source*** | ***CE-marking*** | ***FDA 510(k)*** | ***References*** |
| --- | --- | --- | --- | --- | --- |
| **Clinical software** | | | | | |
| **cPET** Combinostics | Yes (PET-only and PET-MR) | No | Yes | Yes | combinostics.com |
| **Hermia Neurology** Hermes Medical Solutions | Ongoing (PET-only) | No | Pending | Pending | hermesmedical.com |
| **MIMneuro®** MIM | Yes (PET-only) | No | Yes | Yes | mimsoftware.com |
| **NeuroCloud® PET** Qubio | Ongoing | No | Yes |  | qubiotech.com |
| **Syngo.PET** Siemens  Healthineers | Yes | No | Yes | Yes | siemens-healthineers.com |
| **NeuroQ** Syntermed | Ongoing (PET-only) | No | Yes | Yes | syntermed.com |
| **Research tools** | | | | | |
| AmyPET | PET-MR | Yes | N/A | | https://github.com/AMYPAD/AmyPET |
| CapAIBL | PET-only and PET-MR | Yes |  |  | https://doi.org/10.1016/j.neuroimage.2018.08.044 https://doi.org/10.1016/j.jalz.2019.02.005 |
| PNEURO PMOD Bruker |  | No |  |  | https://www.pmod.com/web/?portfolio=centiloid |
| rPOP | PET-only | Yes |  |  | https://doi.org/10.1016/j.neuroimage.2021.118775 |
| SPM12 toolbox | PET-MR | Yes |  |  | https://doi.org/10.1002/brb3.3092 |

**
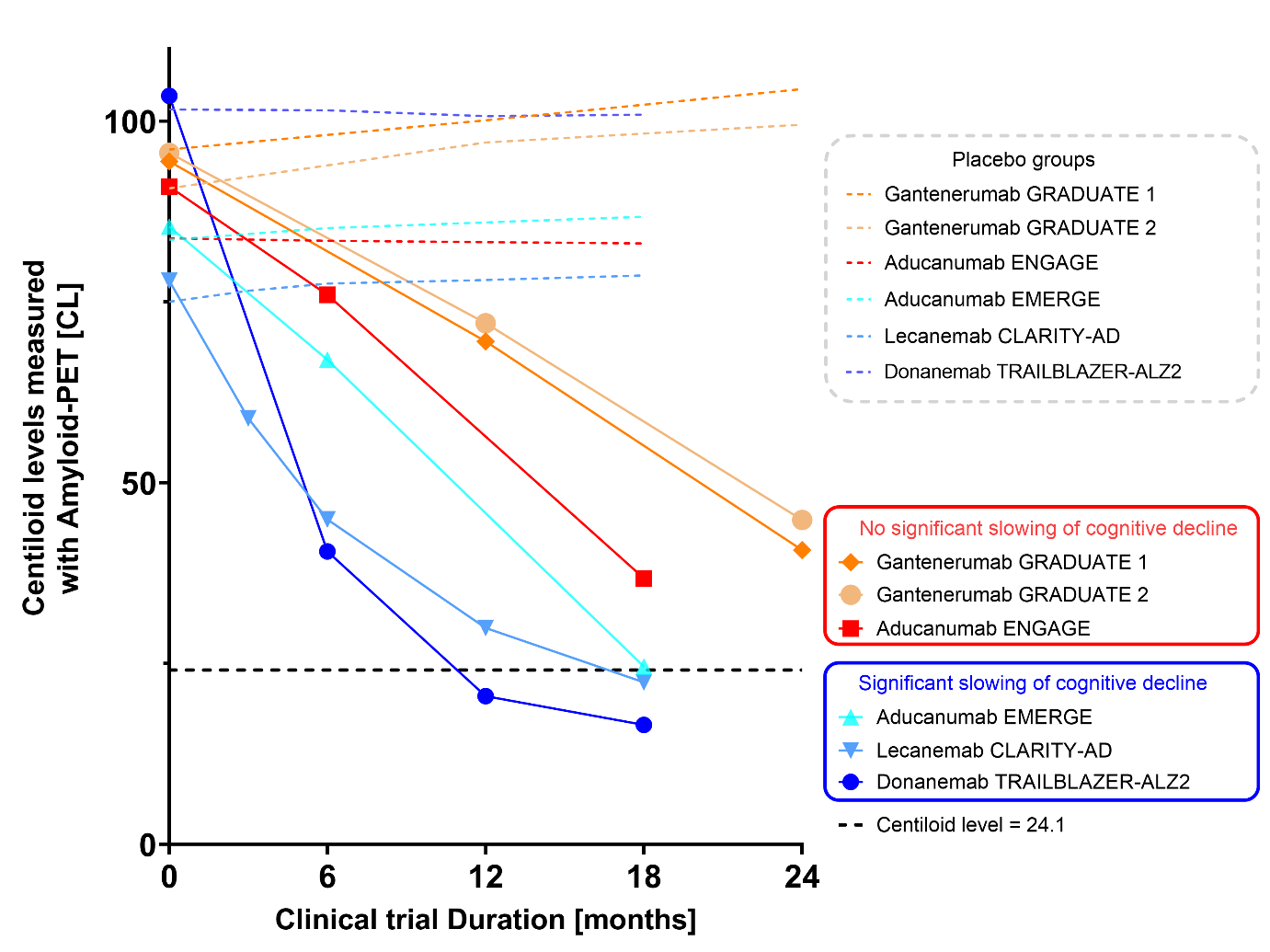
**

**Supplementary Figure-1.** **Amyloid-β removal profiles and placebo groups for Phase 3 trials of aducanumab, donanemab, gantenerumab, and lecanemab as measured by Centiloid.**

Sample sizes in the treatment and placebo arms varied for the individual trials and were at the last visit *N*=614 (treatment) and N=690 (placebo) for donanemab, *N*=210 (treatment) and N=205 (placebo) for lecanemab, *N*=50 (treatment) and N=41 (placebo) (Graduate 1) and *N*=41 (treatment) and N=46 (placebo) (Graduate 2) for gantenerumab, and *N*=109 (treatment) and N=109 (placebo) (Emerge high-dose) and *N*=112 (treatment) and N=124 (placebo) (Engage high-dose) for aducanumab. Data points as reported in Sims et al., 2023; van Dyck et al., 2022; Bateman et al., 2023 and Budd Haeberlein et al., 2022. Line represents 24.1 CL, the cut-off for amyloid-negativity as defined within the GRADUATE 1 and 2 trials and implemented in the head-to-head studies of donanemab vs Aduhelm.


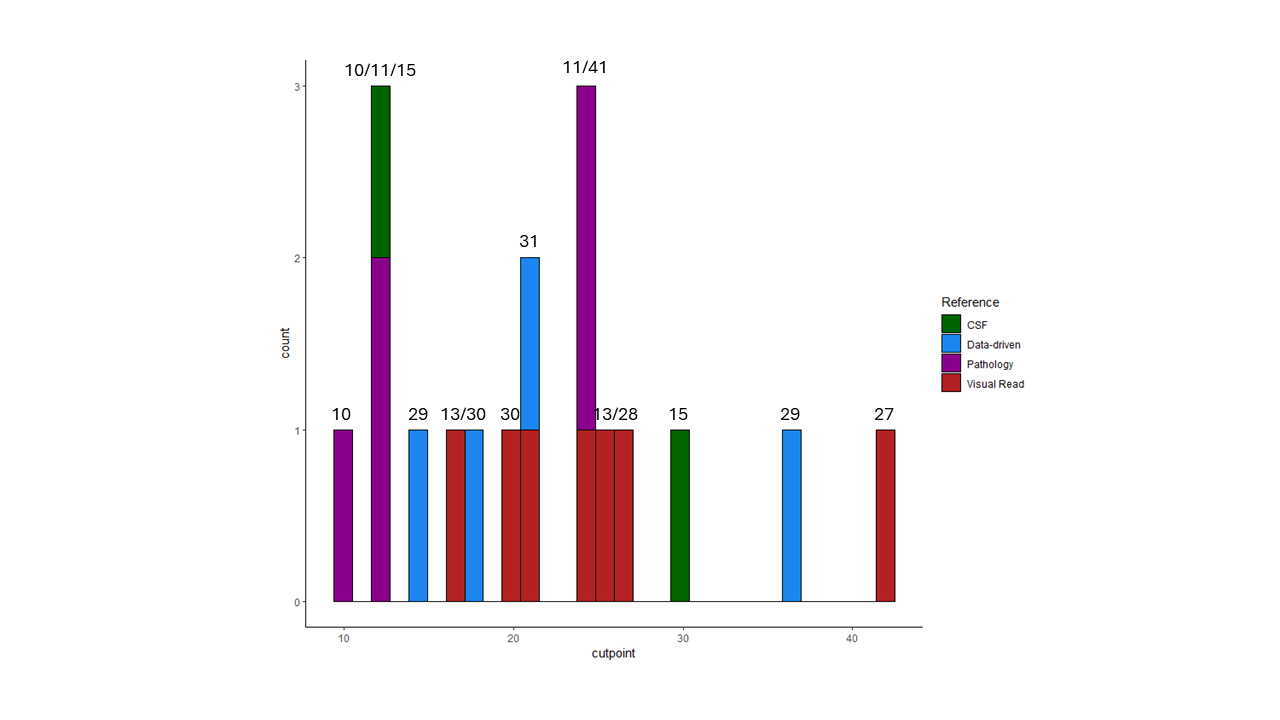


**Supplementary Figure-2. Distribution of amyloid-positive cut-points.**

Please note that cut-points were rounded for comparison. Cut-points are color-coded for the reference data or method used to derive them. Numbers indicate the reference to the papers.

**Supplementary Figure-3. Detailed overview of additional CL cut points used in or derived from different settings**
